# Coffee consumption and CYP1A2 genotype in relation to bone mineral density of the proximal femur in elderly men and women: a cohort study

**DOI:** 10.1186/1743-7075-7-12

**Published:** 2010-02-22

**Authors:** Helena Hallström, Håkan Melhus, Anders Glynn, Lars Lind, Ann-Christine Syvänen, Karl Michaëlsson

**Affiliations:** 1 Research and Development Department, Toxicology Division, National Food Administration, Box 622, SE-751 26 Uppsala, Sweden; 2 Uppsala Clinical Research Center (UCR), University Hospital, SE-751 85 Uppsala, Sweden; 3 Department of Medical Sciences, Section of Clinical Pharmacology, Uppsala University, SE-751 85 Uppsala, Sweden; 4 Department of Medical Sciences, Section of Acute and Internal Medicine, Uppsala University, SE-751 85 Uppsala, Sweden; 5 Department of Medical Sciences, Molecular Medicine, Uppsala University, SE-751 85 Uppsala, Sweden; 6 Department of Surgical Sciences, Section of Orthopedics, Uppsala University, SE-751 85 Uppsala, Sweden

## Abstract

**Background:**

Drinking coffee has been linked to reduced calcium conservation, but it is less clear whether it leads to sustained bone mineral loss and if individual predisposition for caffeine metabolism might be important in this context. Therefore, the relation between consumption of coffee and bone mineral density (BMD) at the proximal femur in men and women was studied, taking into account, for the first time, genotypes for cytochrome P450 1A2 (CYP1A2) associated with metabolism of caffeine.

**Methods:**

Dietary intakes of 359 men and 358 women (aged 72 years), participants of the Prospective Investigation of the Vasculature in Uppsala Seniors (PIVUS), were assessed by a 7-day food diary. Two years later, BMD for total proximal femur, femoral neck and trochanteric regions of the proximal femur were measured by Dual-energy X-ray absorptiometry (DXA). Genotypes of CYP1A2 were determined. Adjusted means of BMD for each category of coffee consumption were calculated.

**Results:**

Men consuming 4 cups of coffee or more per day had 4% lower BMD at the proximal femur (p = 0.04) compared with low or non-consumers of coffee. This difference was not observed in women. In high consumers of coffee, those with rapid metabolism of caffeine (C/C genotype) had lower BMD at the femoral neck (p = 0.01) and at the trochanter (p = 0.03) than slow metabolizers (T/T and C/T genotypes). Calcium intake did not modify the relation between coffee and BMD.

**Conclusion:**

High consumption of coffee seems to contribute to a reduction in BMD of the proximal femur in elderly men, but not in women. BMD was lower in high consumers of coffee with rapid metabolism of caffeine, suggesting that rapid metabolizers of caffeine may constitute a risk group for bone loss induced by coffee.

## Introduction

Caffeine is the most widely used central nervous system stimulant in the world. There are several conceivable health benefits with the intake of caffeine-containing beverages but they can also produce unwanted health consequences. Caffeine increases calcium excretion [1-4] and decreases intestinal calcium absorption [5], with 5 mg net loss of calcium per cup of coffee [1]. A high intake of coffee could therefore also induce loss of bone mineral.

Results from epidemiological studies investigating the relation between coffee consumption and bone mineral density (BMD) in both women [6-14] and men [12,15-21] have been conflicting, which might be explained by differences in sample size, method of data collection and amount of coffee consumed. In addition, it has been suggested that a high caffeine intake is only deleterious for bone health when calcium intake is low [22]. In Sweden, consumption of coffee and thus caffeine intake is high in a substantial proportion of the population, making this setting suitable to study the relation between coffee and BMD and subsequently the risk of osteoporosis.

Several enzymes are involved in the metabolism of caffeine, but the most important is cytochrome P450 1A2 (CYP1A2) [23]. The first step in this metabolic pathway is a N^3^-demethylation, which results in the formation of 1,7-dimethylxanthine, i.e., paraxanthine [23]. A wide variability in CYP1A2 activity between individuals has been observed [24]. Depending on genotype, some individuals are regarded as slow metabolizers of caffeine, while some are regarded as rapid metabolizers [25,26]. There is also a gender difference with men, on average, having higher CYP1A2 activity [27]. To our knowledge, no study has yet investigated how coffee consumption could affect BMD in relation to the rate of caffeine metabolism determined by the genetic constitution of the individuals consuming coffee. However, in a study of coffee intake, CYP1A2 genotype and risk of myocardial infarction coffee was associated with an increased risk of nonfatal myocardial infarction only in participants regarded as slow metabolizers of caffeine [26]. Accordingly, in the present study we hypothesize that the participants' genotype for cytochrome CYP1A2 could modify the relation between coffee consumption and BMD. This is because caffeine exposure of the body will last for longer periods in the "slow" caffeine metabolizers than in the "rapid" caffeine metabolizers. Until now, however, the possibility of modulation by genotype for CYP1A2 has not been considered in studies of coffee consumption and BMD.

The principal aim of this study was to investigate the relation between consumption of coffee and BMD of the proximal femur in a population-based cohort of 70-year-old Swedish men and women. A secondary aim was to study whether the relation between consumption of coffee and BMD in the cohort was modified by the participants' genotype for cytochrome P450 1A2 (CYP1A2).

## Materials and methods

### Subjects

The Prospective Investigation of the Vasculature in Uppsala Seniors (PIVUS) [28] has been described previously [29]. In brief, all 70-year-old individuals residing in Uppsala, Sweden, in 2001-2004 were eligible. Of these individuals, 2,025 were randomly selected and invited to participate within 2 months of their 70th birthday from April 2001 to June 2004. Of those invited, 1,016 (50%) eventually participated in the study. The participants were examined by measurements of blood pressure and anthropometry, blood sampling after an overnight fast, routine medical history and assessment of BMD using Dual-energy X-ray absorptiometry (DXA) as described below. The study was approved by the Ethics Committee of Uppsala University and all participants gave their written informed consent.

### Dietary assessments

Dietary habits were registered in 850 (84%) of the participants. Each participant recorded his or her food consumption during 7 consecutive days using a pre-coded food diary after instructions from a dietician. The pre-coded food diary had been prepared and previously used by the Swedish National Food Administration (NFA) and Statistics Sweden in a food survey of 3,000 households in 1989 [30]. The questionnaire has been validated [30,31]. The menu book included written instructions with an example on how to complete the book. The record sheets started with "day 1" followed by six additional days. For each meal (breakfast, lunch, dinner and snacks), the respondent was asked to specify where and at what time the meal was eaten. The amounts consumed were reported in household measurements or specified as portion sizes according to a photograph showing four portion sizes. Coffee and tea consumption was registered six times daily (breakfast, lunch, supper, between meals and in the evening).

The daily intake of energy, caffeine, alcohol and selected nutrients including calcium, vitamin D and A, was calculated using a computerized program and information about energy and nutrient contents of foods from a database from the National Food Administration that included 1,500 food items, drinks and recipes. Filtered or brewed coffee is the most popular type of coffee in the Nordic countries, while it should be noted that decaffeinated coffee and tea are not typically consumed in the Swedish diet [23]. One cup of filtered coffee (150 mL) was estimated to contain approximately 100 mg caffeine [23]. One cup of tea (200 mL) was estimated to contain about 50 mg of caffeine [23]. No analyses of caffeine content of the consumed coffee and tea were performed.

### Measurement of bone mineral density at the proximal femur

On average, 2 years after the baseline investigation, 898 of 1,016 cohort members agreed to undergo measurements of BMD (g/cm^2^) for total proximal femur, femoral neck and trochanteric regions of the proximal femur by DXA (DPX Prodigy, Lunar corp., Madison, WI, USA). This is the site of the most serious consequences of osteoporosis - the hip fracture [32], which constitutes two main fracture categories: the femoral neck and the trochanteric femoral fracture. When applicable, both extremities were used in the calculation. By triple measurements in 15 participants, the precision error of the DXA measurements of total proximal femur in our laboratory has been calculated to be about 0.7%.

### Genotyping of CYP1A2

A common polymorphism in both Caucasians and Asians is the variation of the nucleotide at position -163 in intron 1 of the *CYP1A2 *gene. The C allele at position -163 in the *CYP1A2 *gene is considered to confer decreased inducibility to the enzyme [24,33,34]. Consequently, carriers of a C allele at this position are regarded as "slow" metabolizers of caffeine [24,33,34]. Enzyme inducibility is increased by a substitution of C with A at position -163 in the *CYP1A2 *gene and homozygote carriers of the mutated allele are considered "rapid" caffeine metabolizers of caffeine. In a previous study, polymorphisms of rs762551 in the *CYP1A2 *gene have been shown to influence the association between coffee intake and myocardial infarction [26]. However, because this single nucleotide polymorphism (SNP) was later not genotyped in HapMap [35], we chose another SNP in HapMap, rs11854147, which is in linkage disequilibrium with rs762551 (R^2 ^= 0.886). The SNP rs11854147 was genotyped at the SNP Technology Platform at Uppsala University, Sweden [36] using the Illumina BeadStation 500GX and the 384-plex Illumina Golden Gate assay (Illumina Inc., San Diego, CA, USA) [37]. The sample success rate was 98.8% and the reproducibility 100% according to duplicate analysis of 2.4% of the genotypes. The genotype distribution was in Hardy-Weinberg equilibrium.

### Statistical analyses

We had the possibility to include 717 genotyped participants with both dietary assessment and BMD measurement in our analysis. All statistical calculations were performed using SAS (SAS 9.1; SAS Institute Inc., Cary, NC). The relation between coffee consumption as a continuous variable and BMD was primarily analyzed by ordinary linear regression models. We further categorized coffee intake by quartiles (0-2 cups/day, 3 cups/day, 4 cups/day and more than 4 cups/day), and the least square means of BMD for each quartile was estimated on the basis of the regression estimates using the General Linear Model (GLM). All estimates were age-adjusted (at time of the DXA measurement) or adjusted by a multivariable model. The multivariable model included age, height, weight, total caloric intake; intakes of vitamin D, vitamin A, calcium, alcohol and tea (all continuous). Categorized variables included in the model were smoking (never, current, former) and levels of leisure physical activity (low, medium, high). Physical activity was divided into light and hard exercise and classified as number of activities for at least 30 min per week. The participant were asked how many times per week he/she performed light (e.g. walking, gardening) respectively hard exercise (e.g. running, swimming) for at least 30 min [38]. Based on the responses to these questions, three physical activity categories were constructed: low, medium, and high. The questions used in PIVUS were similar to the questions used in the Uppsala Longitudinal Study of Adult Men (ULSAM) cohort [39]. The questions in ULSAM have been validated [40].

Our hypothesis that the participants' *CYP1A2 *genotype could potentially modify the relation between coffee intake and BMD was tested in high consumers (both sexes) of coffee (4 cups or more per day). "Slow" metabolizers were defined as participants with genotypes C/T (40.6%) or T/T (10.7%) while "rapid" metabolizers were those with genotype C/C (48.7%). Average multivariable-adjusted BMD values of "slow" and "rapid" metabolizers were compared. To eliminate potential inducing effects of smoking on *CYP1A2 *the analyses were repeated in nonsmoking participants only.

We additionally analyzed whether there existed a difference in adjusted mean BMD values in men and women with a high consumption of coffee (4 cups or more per day) according to their calcium intake: low (<600 mg/day), intermediate (600-1200 mg/day) and high (>1200 mg/day) total calcium intake (including diet and supplements).

## Results

Characteristics of the participants in relation to consumption of coffee are displayed in Table 1. Half of the participants reported consumption of 3 or 4 cups of coffee daily and one fourth reported an intake of more than 4 cups of coffee per day. High consumers of coffee in both men and women had a higher intake of energy and nutrients. However, their body mass index (BMI) was similar to that for low consumers of coffee. Self-reported leisure physical activity was also comparable between categories of coffee consumers, whereas current smoking was more prevalent in both men and women with high consumption of coffee compared with those who drank none or small amounts (0-2 cups) of coffee.

**Table 1:** Baseline characteristics of the participants by amount of coffee consumption at the 1^st ^investigation of the PIVUS cohort^a^

	Men (n = 359)				Women (n = 358)			
**Characteristics**	**Categories of coffee consumption (cups^b^/day)**				**Categories of coffee consumption (cups/day)**			

	**0-2**	**3**	**4**	**>4**	**0-2**	**3**	**4**	**>4**

Number of persons	82	81	85	111	92	110	76	80
Mean age at baseline (years)	72.0 ± 0.8	71.8 ± 0.9	71.8 ± 0.8	72.0 ± 0.8	72.1 ± 0.9	72.1 ± 0.9	72.1 ± 0.8	72.2 ± 0.9
Calcium intake (mg/day)	850 ± 323	949 ± 306	1059 ± 392	1118 ± 365	852 ± 260	912 ± 263	962 ± 267	1011 ± 319
Vitamin D intake (μg/day)	5.7 ± 2.1	5.9 ± 2.0	6.4 ± 2.2	7.1 ± 2.9	5.0 ± 1.8	5.1 ± 1.6	5.8 ± 1.8	5.5 ± 2.1
Vitamin A intake (mg/day)	0.78 ± 0.53	1.0 ± 0.70	0.95 ± 0.64	1.10 ± 0.76	0.78 ± 0.69	0.81 ± 0.53	0.91 ± 0.65	0.84 ± 0.57
Energy intake (kcal/day)	1830 ± 452	1953 ± 421	2102 ± 510	2308 ± 591	1557 ± 404	1698 ± 352	1812 ± 370	1834 ± 457
Weight (kg)	83.9 ± 11.9	80.6 ± 10.3	84.6 ± 14.8	82.8 ± 12.8	68.7 ± 14.5	70.0 ± 13.0	70.2 ± 13.3	69.2 ± 11.8
Height (cm)	175.4 ± 6.0	175.6 ± 5.7	176.3 ± 6.6	175.3 ± 6.8	161.4 ± 6.0	161.3 ± 5.5	161.6 ± 5.2	161.7 ± 5.7
Body mass index (kg/m^2^)	27.3 ± 3.7	26.2 ± 3.3	27.2 ± 4.2	26.9 ± 3.7	26.4 ± 5.5	26.9 ± 4.4	26.9 ± 5.1	26.5 ± 4.4
Alcohol use (g/day)	11.0 ± 10.0	7.7 ± 7.5	9.5 ± 9.7	8.3 ± 8.7	5.0 ± 5.7	4.1 ± 4.6	4.8 ± 4.8	4.1 ± 4.7
Smoking status								
Never	34/82 (41)	42/81 (52)	32/85 (38)	49/111 (44)	49/92 (53)	58/110 (53)	48/76 (63)	37/80 (46)
Current	3/82 (4)	6/81 (7)	5/85 (6)	15/111 (14)	10/92 (11)	10/110 (9)	7/76 (9)	14/80 (18)
Former	45/82 (55)	33/81 (41)	48/85 (56)	46/111 (41)	33/92 (36)	42/110 (32)	21/76 (28)	29/80 (36)
Levels of physical activity								
Low	9/82 (11)	1/81 (1)	4/85 (5)	12/111 (11)	9/92 (10)	5/110 (5)	3/76 (4)	3/80 (4)
Medium	37/82 (45)	41/81 (51)	46/85 (54)	52/111 (47)	46/92 (50)	56/110 (51)	35/76 (46)	39/80 (49)
High	35/82 (43)	38/81 (47)	31/85 (36)	45/111 (41)	37/92 (40)	46/110 (42)	35/76 (46)	34/80 (43)

^a ^All values are mean ± SD (continuous variables) or frequencies (categorical variables). Values in parentheses are frequencies expressed in percent. ^b ^The volume of one cup of coffee is 150 mL

After multivariable adjustment, there was a trend of decreased BMD at the total proximal femur with increasing amounts of coffee consumed (p for trend 0.04) (Table 2). Men who consumed 4 cups of coffee or more per day had a 4% lower BMD at the total proximal femur as compared with men who drank 0-2 cups per day (p = 0.04). This difference was not observed in the female participants. BMD of the femoral neck and trochanteric region of the proximal femur was reduced by 3-5% in men consuming 4 cups or more of coffee per day (femoral neck p = 0.05 and trochanter region p = 0.01 - data not shown). Results for caffeine intake mirrored those for coffee consumption (data not shown).

**Table 2:** Age-adjusted and multivariable-adjusted^a ^bone mineral density (BMD) in the proximal femur (mean and 95% CI) of the PIVUS cohort by amount of coffee consumption

Categories of coffee consumption (cups^b^/day)					
	**0-2**	**3**	**4**	**>4**	**β^c ^(95% CI) per cup** **p for trend**

**All participants (n = 717)**					

	n = 174	n = 191	n = 161	n = 191	
Age-adjusted	0.96 (0.94, 0.98)	0.93 (0.91, 0.95)	0.95 (0.93, 0.97)	0.95 (0.92, 0.97)	0.0006 (-0.0061, 0.0074)
	Reference	p = 0.06	p = 0.59	p = 0.44	p = 0.85
Multivariate-adjusted	0.96 (0.94, 0.98)	0.94 (0.93, 0.96)	0.94 (0.92, 0.96)	0.94 (0.92, 0.96)	-0.0064 (-0.0127, -0.0001)
	Reference	p = 0.15	p = 0.09	p = 0.08	p = 0.04

**Men (n = 359)**					

	n = 82	n = 81	n = 85	n = 111	
Age-adjusted	1.05 (1.02, 1.09)	1.00 (0.97, 1.04)	1.01 (0.98, 1.04)	1.01 (0.98, 1.03)	-0.0054 (-0.0133, 0.0025)
	Reference	p = 0.03	p = 0.07	p = 0.03	p = 0.18
Multivariate-adjusted	1.05 (1.02, 1.08)	1.02 (0.99, 1.04)	1.01 (0.98, 1.03)	1.01 (0.98, 1.03)	-0.0072 (-0.0151, 0.0008)
	Reference	p = 0.10	p = 0.04	p = 0.04	p = 0.08

**Women (n = 358)**					

	n = 92	n = 110	n = 76	n = 80	
Age-adjusted	0.87 (0.85, 0.90)	0.87 (0.85, 0.90)	0.88 (0.85, 0.91)	0.86 (0.83, 0.89)	-0.0043 (-0.0136, 0.0054)
	Reference	p = 0.96	p = 0.72	p = 0.57	p = 0.40
Multivariate-adjusted	0.87 (0.85, 0.90)	0.87 (0.85, 0.90)	0.88 (0.85, 0.91)	0.86 (0.84, 0.89)	-0.0041 (-0.0138, 0.0056)
	Reference	p = 0.91	p = 0.73	p = 0.62	p = 0.41

^a ^Adjusted by age at the BMD-measurement, height, weight, total caloric intake, vitamin D intake, vitamin A intake, calcium intake, alcohol intake, intake of tea (all continuous), smoking (never, current, former) and levels of leisure physical activity (low, medium, high) ^b ^The volume of one cup of coffee is 150 mL^c ^Per cup of coffee

**Figure 1:**
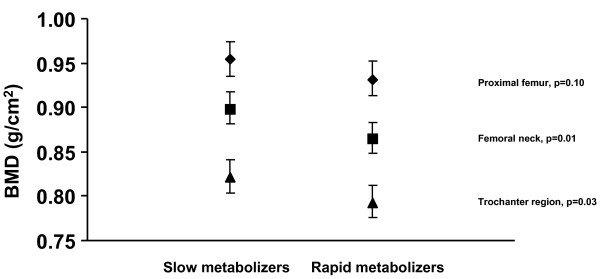
**Mean adjusted BMD (bone mineral density) of the total proximal femur in men and women with a high consumption of coffee (4 cups or more per day) by CYP1A2 polymorphism**. The error bars indicate 95% confidence intervals (CI) and the p-values refer to comparisons between slow and rapid metabolizers at each site. Mean values are adjusted by age at the BMD measurement, height, weight, total caloric intake, vitamin D intake, vitamin A intake, calcium intake, alcohol intake, intake of tea (all continuous), smoking (never, current, former) and levels of leisure physical activity (low, medium, high).

Multivariable-adjusted mean values in rapid and slow metabolizers (men and women) of coffee with a coffee consumption of 4 cups or more per day are displayed in Figure 1. Lower (approximately 2-4%) BMD values were found in rapid metabolizers of caffeine. The differences reached statistical significance at the femoral neck (p = 0.01) and trochanter region (p = 0.03), but not at the total proximal femur (p = 0.10) (Figure 1). Because smoking is known to induce CYP1A2, current smokers (n = 69) were excluded in an extended analysis of the cohort. The results of this analysis remained essentially unchanged in terms of effects upon BMD (data not shown). There were, furthermore, no statistical differences between slow and rapid metabolizers with a low consumption of coffee or between rapid metabolizers with a low consumption of coffee and slow metabolizers with a high consumption (data not shown).

High consumers (men and women) of coffee (4 cups or more per day) with a high calcium intake (more than 1200 mg per day) did not have higher adjusted average BMD values compared with those with high coffee consumption and low (<600 mg/day) or intermediate (600-1200 mg/day) calcium intake (Data not shown). Finally, tea was consumed by 439 participants (about 60%) in the study. Tea consumption was not associated with multivariable-adjusted BMD (parameter estimate per cup of tea -0.0014 (95% confidence interval -0.0106, 0.0078; p = .77).

## Discussion

In this cohort the consumption of coffee was high. We observed a decrease in BMD of the proximal femur in men consuming 4 cups of coffee or more daily. In high consumers of coffee, rapid metabolizers had lower BMD values than slow metabolizers of caffeine. A potential risk group more prone to develop osteoporosis might, thus, have been identified.

The observed decrease in BMD in male high consumers of coffee could be estimated to correspond to an approximately 30% increased risk of hip fracture, which would imply a considerable increase in view of public health [41]. This increased risk might have impact on total osteoporotic fracture health economy. This is illustrated by the fact that the number of hip fractures worldwide in the year of 2000 was estimated to 1.6 million [42]. The global cost for hip fractures is rising and by 2050 it has been estimated to be about 132 billion US dollars [43].

Earlier studies in men [12,15-21] have not observed any statistically significant relation between consumption of coffee and BMD. It should be noted, however, that some of the studies were small [12,15,21]. In most studies [17-21] the exposure was defined as caffeine intake from both coffee and tea. This approach may not be optimal because both beverages contain several other bioactive substances that may modify the effects of caffeine. Furthermore, many of the earlier studies do not clearly state the exposure as amount of coffee or caffeine consumed [15,17,18,20]. When stated, the average intake of coffee/caffeine varied from approximately 200 mg caffeine per day [19,21] or less than two cups of coffee per day [12] to 3 cups per day in one study [16]. In our study, compared to most other studies, mean intake of caffeine and consumption of coffee was higher: 367 mg/day and 3.2 cups/day, respectively.

In the majority of the studies of women no relation between consumption of coffee or intake of caffeine and BMD has been detected [6,7,13,14]. Nevertheless, a weak negative relation between coffee or caffeine and BMD has been observed [9,11,22,44-48] but the relation between BMD and intake of coffee/caffeine has been attenuated by adequate intake of milk/calcium [9,22,47]. Our results do not support these latter findings but few of our participants had a low calcium intake. In general, the studies in women thus provided limited evidence for the existence of a relation between intake of coffee/caffeine and effects on BMD, which is accordance with our results. It should be noted that as in the studies of men, many of the studies in women were small [9,49-57]. In addition, no separate analyses of coffee and tea were carried out in the majority of studies of women [8-10,44,45,48-56,58,59]. Average intake of coffee or caffeine seems to have been low or modest in some studies [10,44,53,55,56], i.e., lower than in the present investigation.

There is evidence for females having lower activity of CYP1A2 than men [27]. With a higher CYP1A2 activity in men, caffeine will be more rapidly metabolized and the concentrations of metabolites like paraxanthine will become higher in relation to the concentration of caffeine. The deleterious effect of coffee consumption on bone may be an effect of caffeine metabolites. Consistent with this theory is that we observed lower BMD among rapid compared to slow metabolizers of caffeine with a high coffee consumption. Moreover, we found lower BMD among male high consumers of coffee but not among such women, an observation that may be explained by higher CYP1A2 activity in men [27]. In addition, we did not find any statistical differences between slow and rapid metabolizers with a low consumption of coffee or between slow metabolizers with a high consumption of coffee and rapid metabolizers with a low consumption. Our results may thus indicate that a certain level of metabolites must be reached in order to observe a negative effect on BMD. There are, however, no published data regarding effects of metabolites of caffeine on BMD. Therefore more studies are clearly warranted in order to investigate possible mechanisms of interactions regarding caffeine intake and CYP1A2 genotype in relation to BMD. How caffeine or its metabolites exert effects on bone can theoretically be explained by other mechanisms than by reduced renal calcium conservation. According to some *in vitro *studies, caffeine may interfere with bone remodelling process. Tsuang et al (2006) [60] suggested that caffeine may have deleterious effect on the viability of rat osteoblasts, which could enhance the rate of osteoblast apoptosis. In addition, Lu et al (2008) [61] has demonstrated that cell viability also decreased in human osteoblasts treated with caffeine in a dose-dependent manner mainly due to apoptosis. Zhou et al (2009) [62] hypothesise, however, that bone marrow-derived mesenchymal stem cells, which are precursor cells of osteoblasts, may be the real target cells of caffeine-induced osteoporosis *in vivo*. However, it remains to be demonstrated whether a mechanism including direct effects of caffeine or its metabolites on cells involved in the remodelling process could be of importance also *in vivo *at dosages of relevance to humans.

It has been demonstrated that both the parent compound, caffeine, as well as paraxanthine, might be teratogenic after administration of very high doses in mice with skeletal malformations as a consequence [63]. Caffeine is cleared more quickly than paraxanthine and 8 hours after caffeine intake, plasma concentrations of paraxanthine levels exceed those of caffeine [64]. With long-term exposure of high doses of caffeine there is substantial accumulation of paraxanthine [65,66]. Paraxanthine has *in vitro *been found to be a potent suppressor of transforming growth factor beta (TGF-β) [67], which stimulates bone formation, and TGF-β deficiency may result in osteoporosis [68]. Interestingly, paraxanthine has been found to be the most powerful pharmacological repressor of hepatocellular TGF-β dependent connective tissue growth factor expression among the drug family of methylxanthines, including caffeine [67]. The major caffeine derivatives, including paraxanthine, have common mechanisms of action, i.e. competitive antagonism of the adenosine interaction with A_1 _and A_2 _receptors. Deactivation of the adenosine receptors, which are expressed in bone cells, can result in reduced bone formation [69].

### Advantages and limitations of our study

To our knowledge, this is one of few population-based studies investigating possible effects of coffee and tea consumption on BMD in both men and women. In contrast to most other studies, the majority of the participants in our study consumed high amounts of coffee. We had a sufficient number of participants to detect even modest associations. An additional strength is that we did not focus on caffeine intake but on the exposure of coffee and tea separately. This distinction may be important because some studies have indicated that consumption of tea could have a positive influence on BMD, which could counteract the negative influence of coffee. Tea consumption in our study was low and adjusted for in the statistical analyses. The possible modification by genotype for CYP1A2 inducibility has not previously been investigated. We also had the possibility to consider several conceivable covariates in the analysis, including nutrients, physical activity behavior and smoking.

This study nevertheless has several potential limitations. In this study we have measured BMD in the proximal femur only. We refrained from including BMD measurements of the spine since spondylosis is common in elderly individuals, and this condition can confound the relative weak association between BMD and coffee as well as the comparison between sexes. As the measurement of BMD was on average performed 2 years after the dietary investigation, the follow-up time was limited. However, the optimal time between measurements of coffee consumption and BMD is currently not known. Nevertheless, it should be noted that earlier studies on skeletal effects by an exposure that affects calcium metabolism indicate a lag period of 2-3 years before a steady state of bone turnover and BMD is reached [70,71].

Statistically significant differences in BMD between high consumers who were rapid metabolizers and those who were slow metabolizers of caffeine were generally confined to the whole study group of both men and women, probably because statistical power was too low to attain statistical significance in the groups of each gender. There were, however, clear tendencies of a lower BMD in high consuming males who were rapid metabolizers. In women the same pattern could also be observed.

Because the exposure measurement was based on a single dietary measurement, there may be some degree of error in the measurement. The 7-day dietary recording used in the present study has been found to be valid [72] and a high reliability of self-reported measures of caffeine consumption has previously been shown [73]. Nonetheless, because no direct measurements of the caffeine content in the consumed coffee and tea were performed, we lack data on the actual intake of caffeine. Still, recall errors of the exposure are known to lead to conservatively biased estimates.

Temporal changes in the consumption pattern of coffee and tea by time might be of importance but this was not assessed since we only determined baseline frequencies of consumption. However, in the Swedish Mammography Cohort [74] it was found that the consumption of coffee among elderly women during 10 years [75] was relatively constant (Personal communication with Dr SC Larsson, Division of Nutritional Epidemiology, The National Institute of Environmental Medicine, Karolinska Institute, Stockholm, Sweden)

## Conclusion

A high consumption of coffee (i.e. 4 cups or more per day) could contribute to a reduction in BMD of the proximal femur in elderly men. BMD was lower in high consumers of coffee with rapid metabolism of caffeine, suggesting that this group of coffee consumers might be at special risk of bone loss.

## Competing interests

The authors declare that they have no competing interests.

## Authors' contributions

The authors' contributions were as follows - HH and KM: designed the study; HH: analyzed the data and drafted the manuscript; LL: recruited the participants, obtained funding, collected data and is principal investigator for the cohort; A-CS: organized the genotyping; HM and AG contributed to the study design and performance, and assisted with the editing of the manuscript and KM revised the manuscript, supervised the study, collected data and obtained funding. All authors have read and approved the final manuscript.
